# Decoupling Charge Carrier Electroreduction and Enzymatic CO_2_ Conversion to Formate Using a Dual-Cell Flow Reactor System

**DOI:** 10.1021/acsomega.4c02134

**Published:** 2024-09-09

**Authors:** Daniel Moreno, Ayokunle Omosebi, Byoung Wook Jeon, Keemia Abad, Yong Hwan Kim, Jesse Thompson, Kunlei Liu

**Affiliations:** † Missouri State University, Springfield, Missouri 65806, United States; ‡ Institute for Decarbonization and Energy Advancement, University of Kentucky, Lexington, Kentucky 40511, United States; § Ulsan National Institute of Science and Technology, Eonyang-eup, Ulju-gun, Ulsan 44919, South Korea; ∥ Department of Chemistry, University of Kentucky, Lexington, Kentucky 40504, United States; ⊥ Department of Mechanical and Aerospace Engineering, University of Kentucky, Lexington, Kentucky 40504, United States

## Abstract

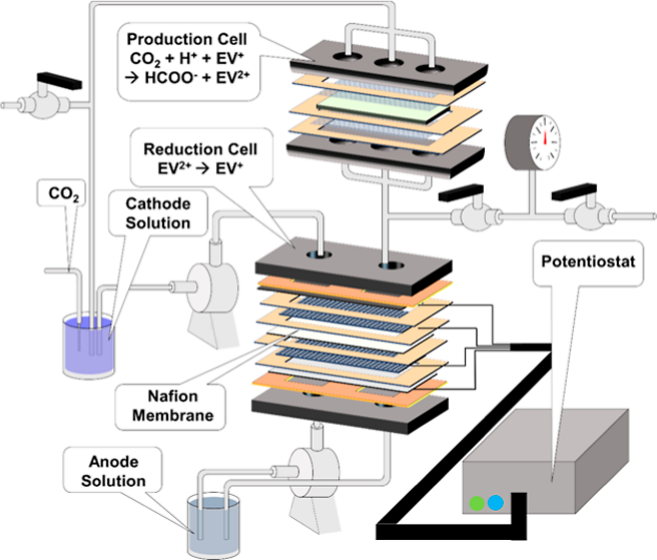

With an efficient atom economy, low activation energy, and valuable applications for fuel cells and hydrogen storage, formic acid (FA) is a useful fuel product to convert CO_2_ and reduce emissions. Although metal catalysts are typically used for this conversion, unwanted side reactions remain a concern, particularly when products are attempted to be recovered long-term. In this study, an enzymatic catalyst is used to enable the selective conversion of CO_2_ to FA, as a formate ion. A dual-cell flow reactor system is used to first reduce a charge mediator electrochemically (reduction cell), which then activates a catalyst to selectively convert CO_2_ to formate (production cell). This approach minimizes enzyme degradation by avoiding direct contact with increased voltages and improves the quantity of formate produced. The system produced 25 mM of formate and reached over 50% Coulombic efficiency. The larger volume of this dual-cell system increases the quantity of formate produced beyond that of a batch cell. Additional design configurations are employed, including a pH control pump to maintain catalyst activity and a packed bed reactor to improve contact of the charge carrier with the catalyst. Both configurations retained higher production and efficiency long-term (∼168 h). The results highlight the challenges of developing a system where many parameters play a role in optimizing performance. Nevertheless, the ability of the system to produce formate from CO_2_ demonstrates the potential to improve upon this configuration for a variety of electrochemical CO_2_ conversion applications.

## Introduction

While research and development of renewable energy continue to rise, a considerable number of sources will still come from fossil fuels, and with this comes atmospheric CO_2_ emissions. The CO_2_ can be converted into other valuable resources to decrease such emission products, including methane, ethylene, methanol, and ethanol.^1−4^ Interest in formic acid (FA) has grown in recent years, primarily for its use in fuel cells and liquid hydrogen storage.^5−7^ Furthermore, FA is unique in that the activation energy and water usage for CO_2_ conversion are minimal compared to other products.^8^

The Kemira process^9^ is the state of the art for converting CO_2_ into FA and is usually energy intensive which can further contribute to emissions. The Kemira process also involves additional intermediate steps in which methanol, already a valuable product, is reformed and then reacted with carbon monoxide. The challenge with the Kemira process lies in the high temperatures and pressures that are involved in the required conversions. An alternative to the Kemira process is to convert the CO_2_ into formate electrochemically and then converting to FA via techniques such as ion exchange (IEX).^10,11^ The electrochemical route for formate (referred to as such henceforth) production can circumvent the need for elevated temperatures and pressures while providing the capability for negative emissions.

Despite the advantage of using less energy, product selectivity remains a challenge for electrochemical conversion to CO_2_.^3,12^ Electrochemical CO_2_ conversion typically uses metal catalysts, the most popular of which are lead, tin, and bismuth. The metal catalyst used will affect the products formed, such as carbon monoxide, hydrogen, and higher-order carbon products.^13,14^ However, the electrochemical conversion of CO_2_ can be difficult to control and often generates unwanted side reactions.^13−15^ Some studies have reported efficiencies approaching 100% for different types of catalysts, but these are usually short-term.^16,17^ Only a limited number of long-term experiments have been reported,^18^ and these are complicated by more different designs involving anion/cation exchange membranes and alternative materials, which can contribute to a less cost-effective design. Longer-term stability remains a challenge, and protonation on the catalyst can expose a vulnerability to unwanted reactions such as hydrogen evolution, and thus form additional products.^19^ Enzymatic catalysts have been found to produce high yields of the desired products^20,21^ and can operate reliably at near-ambient temperature and pressure conditions. Using an enzymatic catalyst will still require additional considerations, most notably the operating temperature and pH ranges for the enzyme activity as well as stability.^22−24^

The process of using an enzymatic catalyst to convert CO_2_ to formate had been demonstrated previously using a small-scale batch reactor.^25−27^ This process involved the use of ethyl viologen (EV) as a charge carrier in the cathode, which would electrochemically reduce at a sufficient voltage (∼−0.7 V vs Ag/AgCl) to an EV^+^ species. Then, the additional charge on the EV would be used in the enzyme to produce formate, enabling the EV^+^ to reoxidize and be used in the next cycle as EV^2+^ once again. To ensure that the pH remained at suitable levels for the enzyme (6.3–6.8), a carbonate buffer would serve to maintain these levels in the cathode. For the anode, a platinum wire was used (due to high corrosion resistance) and submerged in a sulfuric acid solution, serving as a source for protons to convert CO_2_ into formate via the Nafion membrane separating the two chambers. In the short term, the Coulombic efficiency (CE) could approach 100% if the optimal charging voltage was used. However, when scaled up to a larger volume, the batch reactor cannot effectively produce formate at a continuous rate, as accumulation in the batch cell could alter pH and hinder further production, and current densities will be too low to achieve desirable formate production rates.

Electrochemical CO_2_ reactors are often scaled using a flow cell to increase production quantities.^28−30^ The flow reactor, which the authors have previously documented,^31^ decouples the electrochemical charge carrier reduction and subsequent catalyst interaction into two separate processes, each in their own unit cell. To the best of the authors’ knowledge, such a study on a flow cell has not yet been done with this unique configuration. If done in a flow cell configuration, then the system would have two primary benefits: (1) the product catalyst in the production cell would be protected from high overpotentials in the electrode, and (2) the configuration would greatly reduce the chance of fouling deposits from the enzyme accumulating in the reduction cell and subsequently weakening its performance.

This work presents the approach, design/system improvements, and challenges associated with the implementation of an enzymatic catalyst in a decoupled dual-cell flow reactor, including a reduction cell for charge mediator reduction and a production cell for formate production via enzymatic electrocatalysis. The catalyst, formate dehydrogenase 1 from *Methylobacterium extorquens* AM1 (MeFDH1), was prepared using an affinity tag to bind to agarose beads. By using a flow system in lieu of a batch cell, there are several considerations for system operation.^32^ For instance, at the anode, carbon cloth electrodes coated in platinum–iridium (Pt–Ir)^33−35^ are used instead of a larger platinum wire/mesh due to cost considerations and long-term stability. Pt–Ir is generally considered to be a state-of-the-art anode. Typically, the batch cell expects an operating range of −0.75 V vs Ag/AgCl, and in the absence of an applied current, the reduced EV^+^ would eventually reoxidize to EV^2+26^. In the flow system, a much higher operating voltage may be required due to voltage loss during charge mediator transit between the reduction and production cells.

A corresponding evaluation focused on the effect of the location in the flow system on the measured quantity of formate produced. This study also examined whether constant current or constant voltage would be more beneficial for maintaining system performance and efficiency long-term. In a third study, a pH control pump with a potassium hydroxide (KOH) buffer was employed to offset the influx of protons from the acidic buffer with the aim of retaining enzyme stability to maximize formate production and efficiency. The studies were limited by only a finite supply of catalyst; therefore, the experiments run had to be carried out strategically to make the most of their use and provide meaningful results. Here, instead of only the cathodic voltage or system current, overall cell voltage was employed as a constraint to attempt to improve long-term stability. Additionally, this study replaced the membrane production cell with a packed bed reactor to improve contact between the reduced charge mediator and the enzymatic catalyst in the cell to maximize formate production.

## Experimental Section

**Figure 1:**
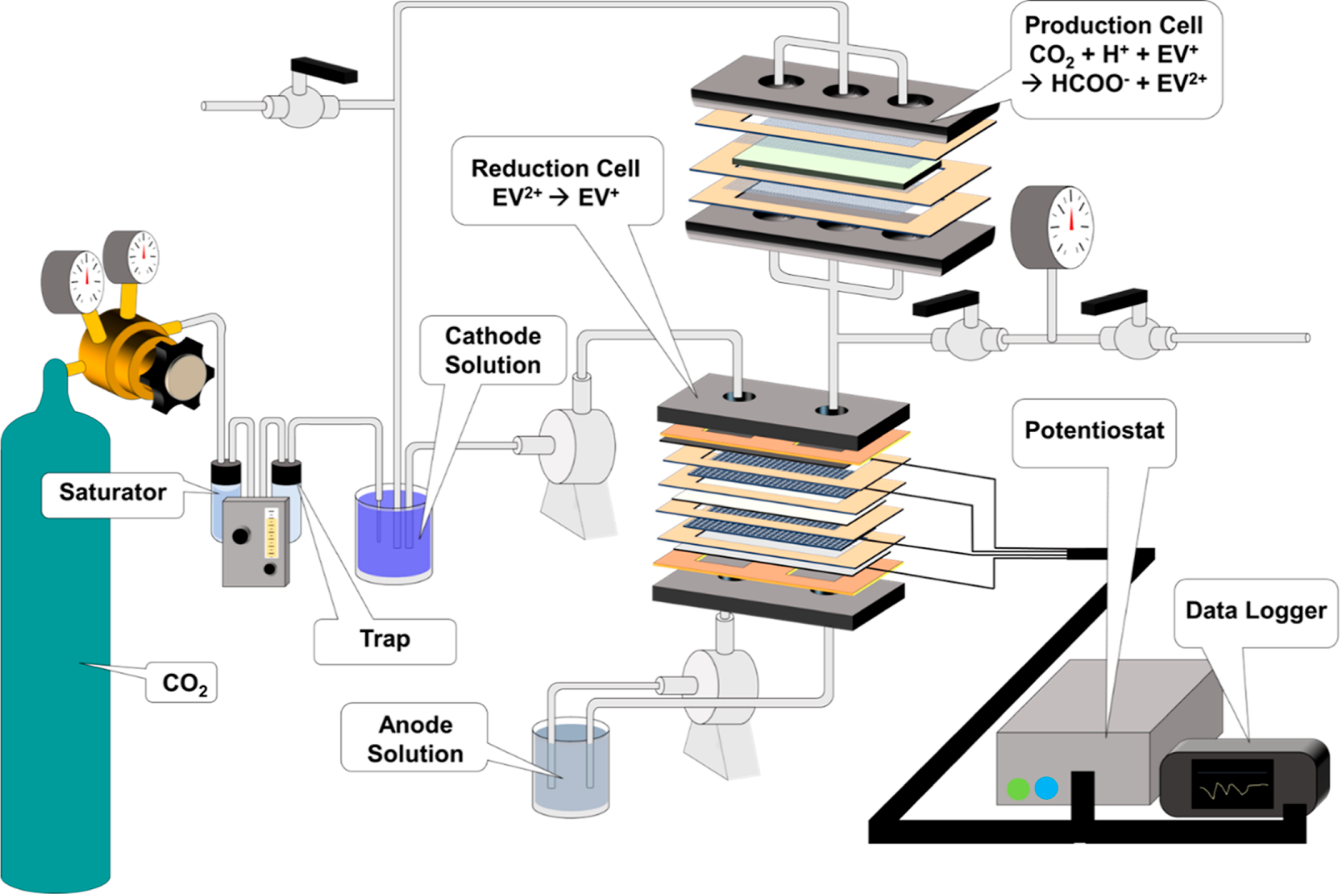
Schematic of a dual-cell flow system indicating individual components of both cells and locations in the reduction cell where voltage values were measured.

The flow system initially considered in the study involved two cells consisting of stainless steel end plates hand-tightened with sufficiently shaped compressed gaskets (Figure 1). The contact area in which the solution could interact with either the electrodes (reduction cell) or enzyme (production cell) was approximately 33 cm^2^ in both cells. In the reduction cell, a Nafion 115 membrane separated the anode and cathode two streams. The anode side used a Pt–Ir-coated electrode (average loading of 0.7 mg/cm^2^ on carbon) and circulated a 100 mM aqueous H_2_SO_4_ solution. The dissociated protons from the sulfuric acid replenished by the oxygen evolution reaction serve as a proton source through the Nafion membrane and travel to the production cell to react with CO_2_ and EV^+^ in the enzyme to produce formate. While some protons could be produced from water splitting, multiple previous studies^36−38^ have employed a sulfuric acid–based anolyte. At all times outside of the reduction or production cells, charge neutrality is conserved by the presence of other species, including HCO_3_^–^, CO_3_^2–^, OH^–^, Cl^–^, HCOO^–^, K^+^, H^+^, EV^2+^, EV^+^, EV_2_^2+^, and EVH^+^. Therefore, the protons can remain in the solution into the production cell, although this will alter the concentration of some other ionic species.

The cathode side used a carbon felt electrode, commonly used in batteries,^39,40^ and a 200 mM potassium carbonate (KHCO_3_) buffer. Using the carbon felt electrode increased current density on average by over 10× for the same applied voltage (Figure S1), which may result in larger formate production rates over traditional copper electrodes that typically produce a wide array of other products. Both cathode and anode solutions were initially filled with a 100 mL volume. The cathode volume doubled to 200 mL when a pH buffer was employed to balance the proton flux from the anode, which diluted the charge mediator/carrier concentration. The charge mediator used in this study is 10 mM EV; other types of viologens such as methyl viologen^41,42^ have been used for enzymatic electrochemical conversion of CO_2_ in previous studies, but for this particular enzyme, EV was found to work best.^26^ The pH buffer was employed only during the third set of testing when the packed bed reactor was present.

**Figure 2:**
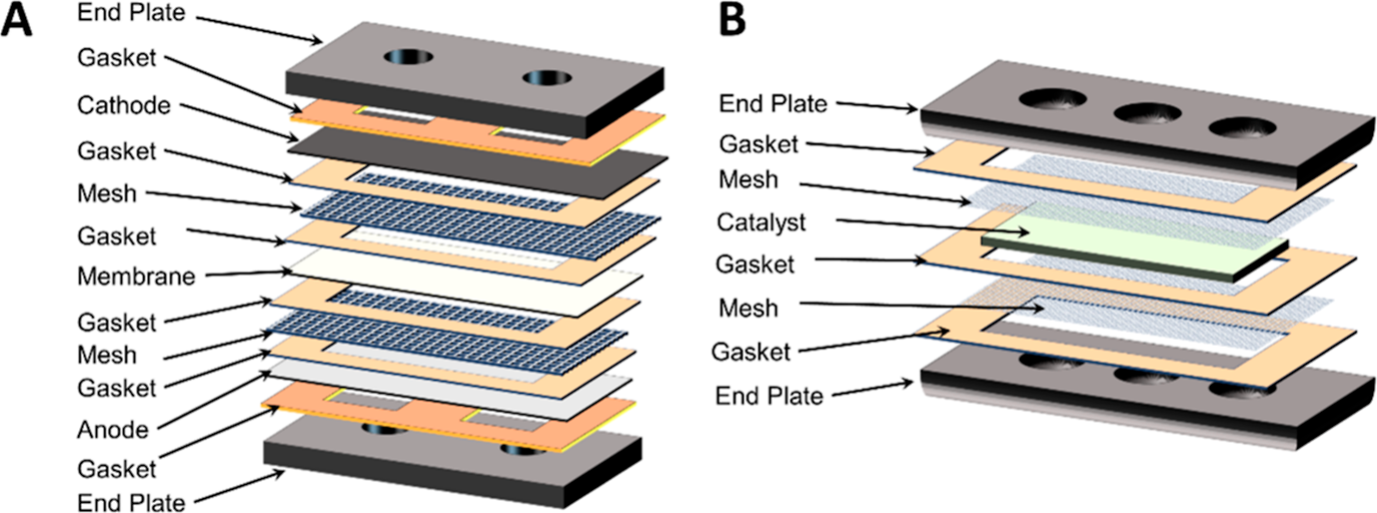
(A) Components of the reduction cell and (B) components of the production cell.

In the reduction cell (Figure 2A), the anode solution was circulated on the bottom side and the cathode at the top to minimize cathode transit time between the two cells. Unless otherwise stated, the default flow rate used during most experiments was 10 mL/min. The production cell (Figure 2B) was essentially a bioelectrochemical cell, with no external wiring connections, and served to house the catalyst. The catalyst would receive reduced EV^+^ from the reduction cell, along with CO_2_ and H^+^, to produce HCOO^–^, converting the reduced EV^+^ back to EV^2+^ in the process. The production cell was placed at an elevation higher than that of the reduction cell and operated in flow-through mode. From the production cell, one pathway would be to separate the formate from the solution and perform an additional protonation step using available technologies such as IEX to produce a purified FA stream.^10,11^

**Figure 3:**
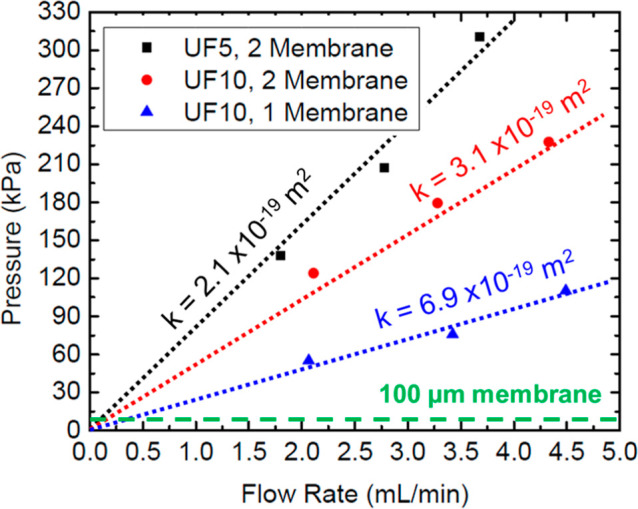
Flow rate vs pressure for different membranes. Pressures are measured as gage pressures.

The pump pressure was used to oppose gravitational effects, which in this configuration would ensure that the catholyte would maximize its contact with the enzyme in the production cell. Initially, microfiltration membranes were used, but they resulted in high pressure increases in the system even at small flow rates (Figure 3). Darcy’s law was used to relate the flow rate and pressure
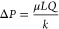
1where μ represents the fluid viscosity, *L* is the length through which a pressure increase is expected, and *Q* is the flow rate. The permeability constant, *k*, was determined from the slope value shown in Figure 3. All values obtained were on the order of 10^–19^ m^2^, in line with impervious surfaces such as granite.^43^ Such results verified the difficulty of using high filtration membranes, even for water with a viscosity of approximately 1 mPa·s. The size of the immobilized agarose beads attached to the catalyst (Table S1) enabled it to be used in a much larger mesh, which kept the overall system pressure at more manageable levels. Ultimately, it was determined that the catalyst was large enough to be contained in a 121 × 121 mesh membrane (McMaster-Carr).

The CO_2_ reduction catalyst in the production cell was formate dehydrogenase 1 from *M. extorquens* AM1 (MeFDH1). An affinity tag was used to immobilize the enzyme by binding it onto agarose beads. Kinetic studies determined the enzyme’s most favorable operating conditions, suggesting that temperatures above ambient would decrease activity. An Econo-Column (Bio-Rad) enabled that the enzyme detached from the beads, where UV–vis spectroscopy could be used to confirm the enzyme’s optimal pH range of 6.3 < pH < 6.8. The enzyme samples were preserved in a MOPS (3-(*N*-morpholino) propanesulfonic acid) buffer and stored at 4 °C. The approximate enzyme activity used in the experiment was 6.7 μM, or approximately 200 U.

The cathode solution initially consisted of 200 mM potassium bicarbonate (KHCO_3_) buffer with 10 mM EV. To minimize the contribution of dissolved atmospheric molecular oxygen present in the cathode solution, a 5 mM scavenger (sodium thiosulfate) was also employed. The scavenger served to decrease the contribution of the current for unwanted reactions and therefore improves the CO_2_ to formate conversion efficiency.^26^ The EV charge carrier was reduced in the reduction cell and then reoxidized in the production cell upon interaction with the catalyst to produce formate. The catalyst’s associated reaction has been described previously^26^ and the subsequent reaction taking place in the production cell is shown in Figure S2. This formate was released by the catalyst back into the bulk solution, where liquid samples could be drawn to determine the quantity produced. Samples were analyzed with ion chromatography (IC), mixed with a 6 M sulfuric acid solution, to prevent the produced formate from reoxidizing. Samples in the flow system were collected at intervals sufficient to evaluate the formate production changes over time.

Voltage was applied by using a Gamry REF-600 potentiostat. The system pressure was measured by using an Omega PX-409 transducer. System voltages, along with the pH, were also recorded using an external MIDI GL220 data logger. A pH buffer has also been implemented to offset pH drops in the system, which could denature the enzymes, either due to the increased presence of protons in the cathode side or production of formate, due to its low p*K*_a_ number. The pH control system implemented is detailed in the Supporting Information (Figure S3).

**Figure 4:**
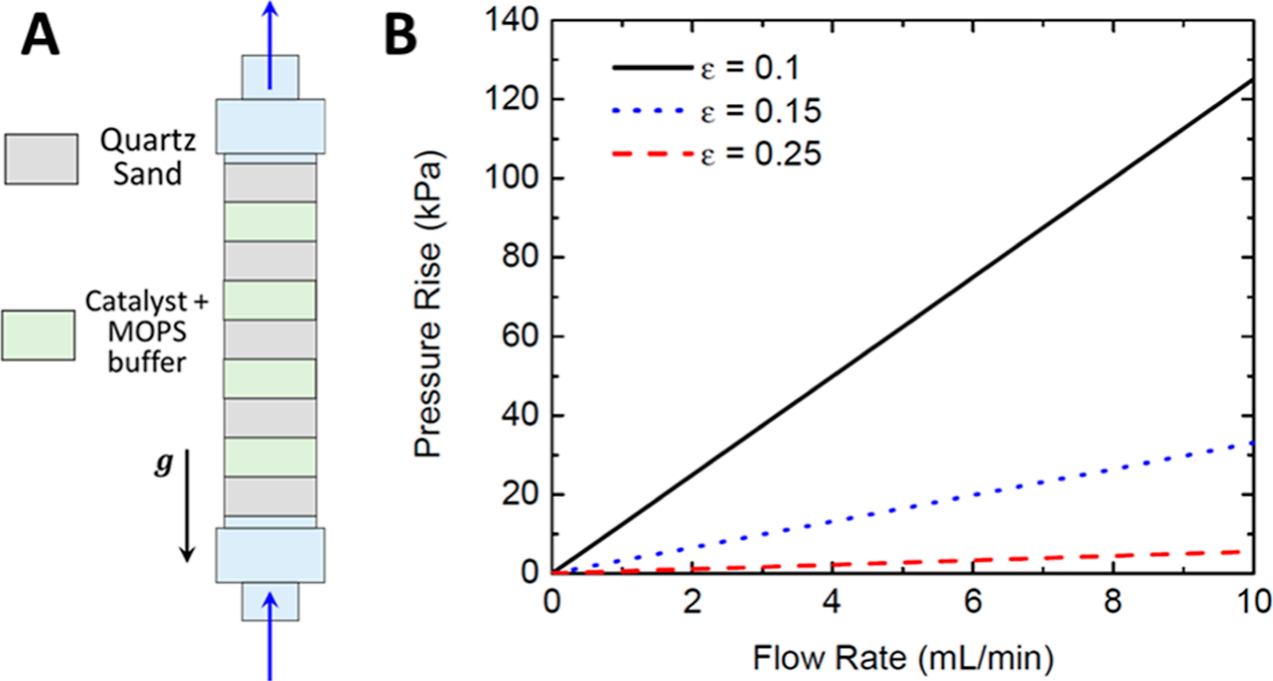
(A) Schematic of packed bed reactor design. (B) Predicted pressure rise as a function of the flow rate and porosity as predicted by the Ergun equation. Sand particle diameter is 0.3 mm with a packed bed length of 11 cm.

The second set of experiments sought to enable better contact with the EV in the production cell to produce formate. Here, the production cell in Figure 1 was replaced with a packed bed column (Figure 4A). This column served a similar function as the production cell, with the catalyst secured through the same 121 × 121 mesh membrane. The catalyst was embedded between volumes of sand to better distribute contact with the packed bed. To determine the dependence of pressure with sand packing on hydrodynamic conditions, the Ergun equation^44^ was used
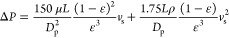
2where μ again represents fluid viscosity, *L* is the length of the bed, *D*_p_ is the packing spherical diameter, ε is the bed porosity, *v*_s_ is the velocity of the fluid entering the bed, and ρ is the fluid density. *D*_p_ was obtained as 0.3 mm using known properties of the packing sand used, while porosity was obtained using the void/pore volume fractions. When the enzyme was included in the system, this porosity value was estimated at 0.1, but other values are included for reference. It should be noted that the variation in pressure with the flow rate appears entirely linear; we attribute this to the extremely small velocities obtained in the system through the packed bed (reaching no more than 1 mm/s, corresponding to entirely laminar flow). The Ergun equation predicted pressure increases to over 200 kPa (Figure 4B). This result is coupled with the initial equation based on the membrane selected and suggests that, coupled with a microfiltration membrane, porosity should be kept high enough to prevent overpressurizing the system. Thus, if system pressure is minimal, pumping energy for the system will be low and thus not as major a concern when considering energy costs for dual-cell configuration long-term. Aligning with Figure 3, this confirms that pressure and corresponding energy requirements for the system are decreased compared with microfiltration.

**Figure 5:**
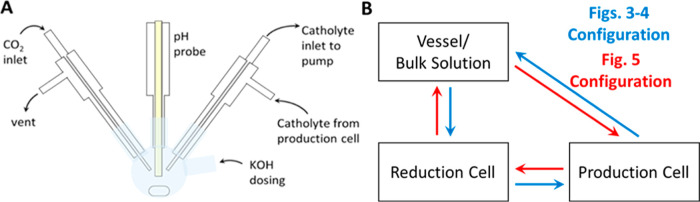
(A) Schematic depicting vessel for cathode solution to accommodate dosing of KOH base. (B) Illustration of flow system components in order through which solution flows. In the clockwise configuration, the cathode solution is pumped first into the production cell, then into the reduction cell, with the storage vessel thus capable of measuring pH immediately before entering the production cell. This configuration was only used in the third set of experiments when the packed bed reactor was present (effectively in place of the production cell).

For the pH control, the system was modified to set the pH upon entering the production cell (Figure 5). KOH would be dosed into the system until the pH reached a desired range of between 6.3 and 6.5.

During optimal operation, the following reactions are expected to occur within the system:

**Anode:**Oxygen Evolution/Proton Formation:
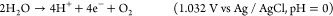
3**Cathode, Reduction Cell:**First Reduction of EV:
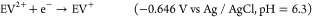
4**Cathode, Production Cell:**Reoxidation of EV:
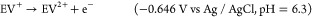
5CO_2_ to Formate:
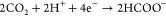
6While the initial buffer (KHCO_3_) is alkaline and purging the system with CO_2_ decreases the pH, the pH remains >6 and the product is formate. Additional, unwanted side reactions may also take place on the cathode side of the reduction cell:Second Reduction of EV:
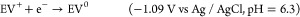
7Dimerization of EV:
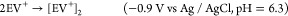
8Hydrogen Evolution:
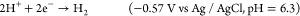
9

Dimerization of EV, however, has been shown to be minimal, particularly when compared with other charge carriers such as methyl viologen.^45^ Hydrogen, while also a desired product in electrochemical fuel production, is neither analyzed nor captured in this study.

The CE of the system is calculated by relating the molar formate production in the system to the total electronic input. As a percentage, this value is expressed as
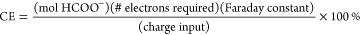
10where the number of moles of formate produced is found by taking its concentration and multiplying by the solution volume in the system. The electrochemical conversion of CO_2_ into formate requires 2 electrons (eq 6). Charge input is given in Coulombs
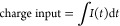
11where *I* represents the input current (in *A*). For eq 10, an absolute value of this quantity is taken since the applied current is negative, typically consistent with the system potential.

## Results and Discussion

### Effect of Sampling Location

Formate samples collected for IC analysis were typically extracted from the bulk solution (shown in Figure 1A, and cathode solution in Figure 1). However, it was suspected that due to the large volume in the system on the cathode side and not all components would be evenly mixed, it may not represent the true quantity of formate produced by the system. This true amount of formate produced would occur directly at the production cell exit. In a large facility when this process is scaled up and run in a continuous/single-pass mode, formate would be extracted at this stage and not immediately mixed back into the bulk solution. A separate study was conducted to determine the effect of sampling location for formate concentration analysis. This was done by inserting an additional valve in between the production cell and bulk solution to immediately extract the outlet from the production cell. This study collected two sets of formate samples in a flow cell experiment: one within the bulk solution, the other immediately at the exit of the production cell, where the formate would eventually be extracted from the solution upon scale-up.

**Figure 6:**
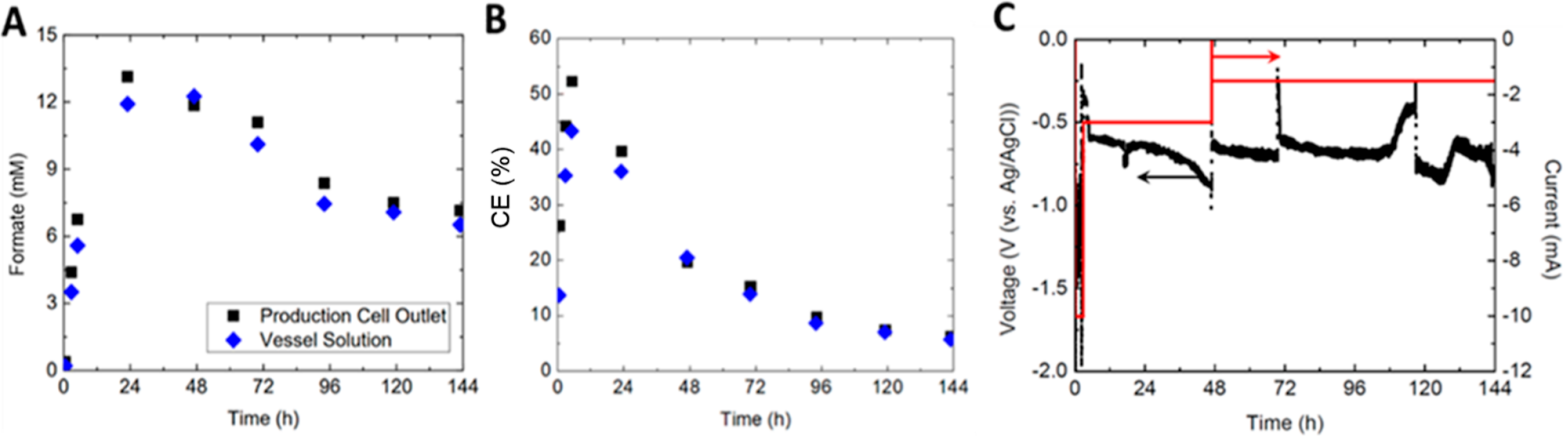
Results obtained for the flow cell with data collected at two different locations in the system. (A) Formate, (B) CE, and (C) applied cathode voltage in the system. Here, the cell was configured according to Figure 1. Applied current used was −10 mA (0.3 mA/cm^2^) over a 200 mM KHCO_3_ catholyte with 10 mM EV and a 1 mM H_2_SO_4_ anolyte.

In this test, the current was initially kept constant to accelerate formate production and maximize system efficiency, as shown in eq 10. Applying a constant current has the benefit of retaining a steady-state efficiency due to the linear charge increase, provided formate production can similarly be maintained. With an applied current of −10 mA (0.3 mA/cm^2^), the cathode voltage eventually grew to nearly −2 V and needed to be subsequently relaxed to ensure that performance metrics of flow rate and efficiency could be maximized, at least for some time. When looking at the amount of formate produced, with one exception, values in the production cell outlet were higher, by up to 20% (Figure 6A). The peak conversion rate of formate shown in Figure 6A, approximately 2 mM/h, is comparable to values previously reported for enzymatic electrochemical conversion to CO_2_.^42^ The current densities are conversely lower due to the larger exposed cell area, but the primary goal here is to ensure that substantial formate production rates and efficiencies can be held long-term.

**Figure 7:**
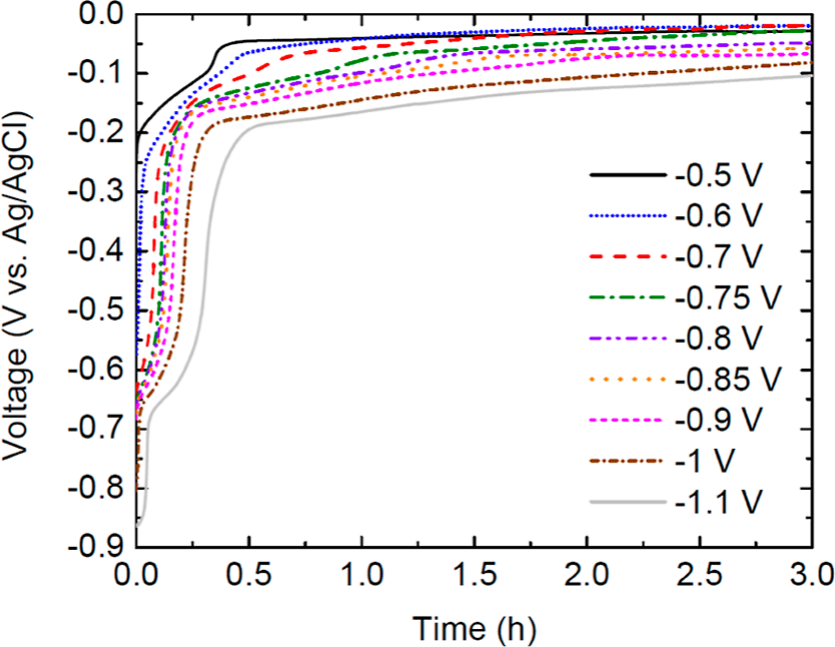
Experimental decay of voltage as measured in a batch cell, initially charged for 1 h. This experiment was run with variable applied voltages using 200 mM KHCO_3_ buffer in a cathode with 10 mM EV and 100 mM H_2_SO_4_ in the anode.

As only formate production was affected by sampling location, efficiency increases followed similarly and allowed values to peak at 50% (Figure 6B). However, efficiency would decrease rapidly following the 24 h period, even when lowering current. The challenge would then be on whether efficiency could be retained in such a system. One area for improvement would be on retaining the high voltage as higher efficiency values returned when the voltage was highest (Figure 6C). This is justified by the transit time between the two cells, which can cause a substantial decrease in the voltage in the production cell (Figure 7), even at low values. Therefore, in addition to the sampling location, it is concluded from this initial study that increased voltages would not be as substantial of an issue; in fact, they would likely be necessary.

Energy efficiency (EE) was also evaluated,^17,46^ using the known Gibbs free energy of liquid FA and comparing it against the electrical energy input, influenced by both voltage and current. Corresponding with Figure 6B,C, the EE reached peak values of over 70%, with an over 10% increase when samples were collected at the production cell outlet (Figure S4).

### Effect of the Applied Voltage

**Figure 8:**
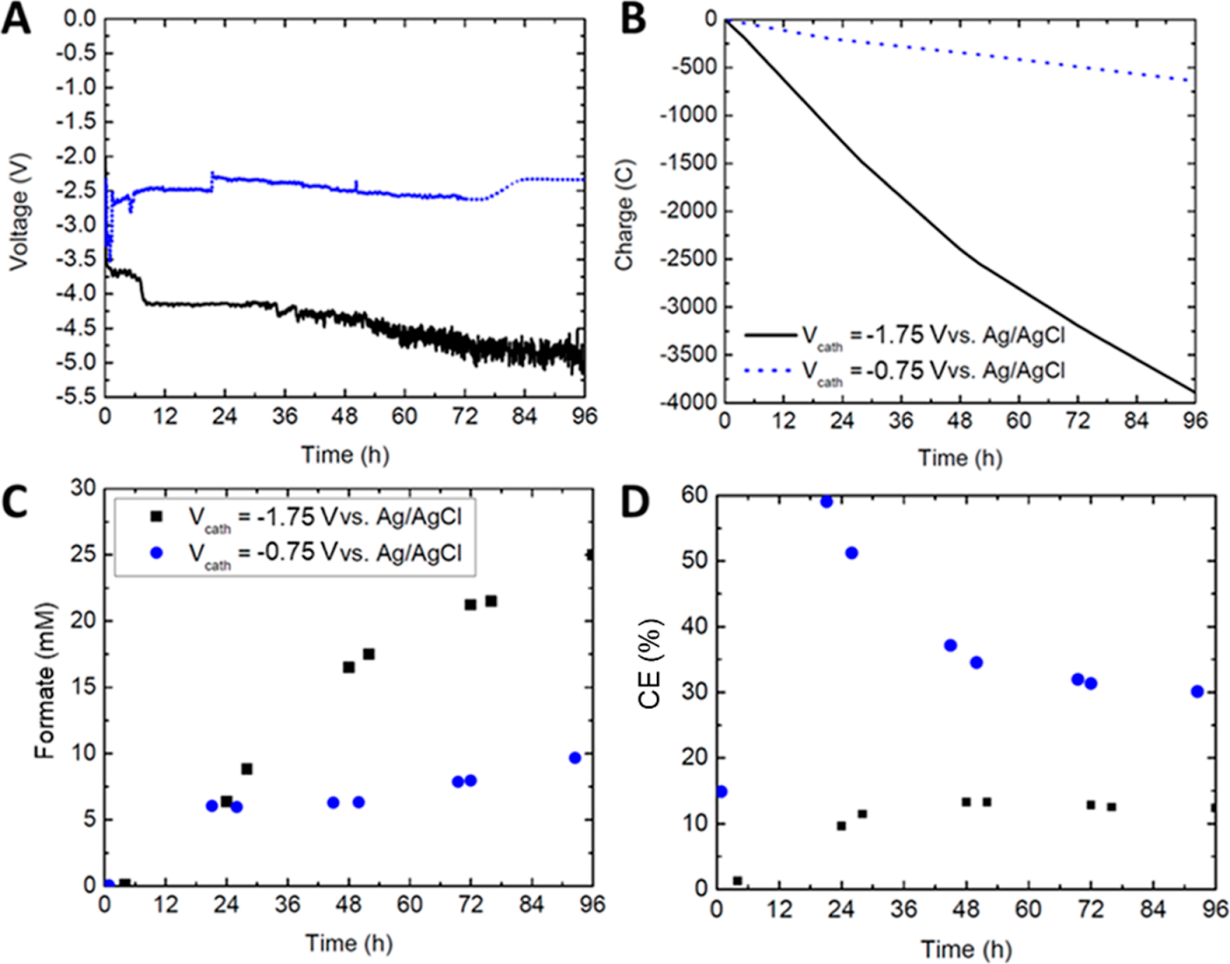
Effect of the charging cathode voltage on flow system performance. (A) Total cell voltage, (B) accumulated input charge, (C) FA produced, and (D) CE. Both tests used the same electrolyte compositions on both cathode (200 mM KHCO_3_ buffer with 10 mM EV charge carrier) and anode (100 mM H_2_SO_4_), and use the configuration shown in Figure 1.

Initially, the effect of the cell voltage in the flow system was examined. To facilitate formate production, a larger voltage (−1.75 V vs Ag/AgCl) was initially tested in the cathode to compensate for the voltage loss between the two cells. The −0.75 V vs Ag/AgCl test was kept as a control here. With the higher cathode voltage used, the total cell voltage magnitude did not increase above 4 V for the first 20 h (Figure 8A). The residence time in the reduction cell (approximately 20 s) was small enough that over-reduction to EV^0^ did not occur, even at the greater applied voltage. The lower voltage test at −0.75 V vs Ag/AgCl still produced formate, despite some reoxidation between the two cells. This indicated that operating in the predicted range (−0.75 to −0.85 V vs Ag/AgCl) could still be sufficient to retain reduced EV^+^ in the reduction cell.

As expected, the −0.75 V test maintained a lower total voltage magnitude of approximately 2.75 V. With the −1.75 V applied, the total cell voltage exceeded 4 V after 8 h, possibly indicating an instability on the anode side due to the excess voltage on the Pt–Ir electrode (as the cathode voltage was held constant) and highlighting the requirement for a geometrically larger or more catalyst-loaded anode. Deposition of the accumulated EV^0^ on the membrane surface could also be another reason for the weakened performance. Nevertheless, as platinum electrodes typically exhibit strong corrosion resistance,^47^ both experiments continued for 96 h.

With −1.75 V applied, the total cell current was increased by nearly 6×, which improved formate production but decreased efficiency due to the accumulated charge (Figure 8B). Formate production peaked at 25 mM with −1.75 V, but it was only 10 mM when charging the cathode at −0.75 V (Figure 8C). Furthermore, when experimenting long-term, formate production decreased to only around 0.3 mM/h at most even though production could be sustained. The formate production values, while below those of the batch cell by nearly an order of magnitude, occurred in a solution with 10× the batch cell volume, resulting in an increase in the total moles of formate produced. Here, the −1.75 and −0.75 V tests would yield approximately 2.5 and 1.3 mmol, respectively. The latter case would demonstrate an improvement in the performance, which would still exceed the batch cell maximum of 2.3 mmol.

For the efficiency values, the tests had trended in opposite directions over time. With −0.75 V, the test started significantly higher but then decayed, peaking above 50% and stabilizing at just over 30%. In the −1.75 V case, efficiency increased over time and peaked at just below 15% (Figure 8D, black). It can be concluded from these results that formate production at higher voltages is insufficient to overcome the higher charge accumulated, weakening the efficiency. Thus, a trade-off between formate production and efficiency exists depending on the desired quantity to be maximized.^26^ EE followed a trend similar to that of CE (Figure S5), with values up to 8× higher when the cell was charged more slowly, corresponding with the voltage decrease seen in Figure 8A.

### pH Control Pump + Packed Bed Reactor

Enzyme availability is limited due to the higher volume needed in the flow system and has been often cited as a challenge in enzyme electrosynthesis.^48^ Proper control of pH was therefore needed and implemented as a means of preserving enzyme longevity for each experiment. Another option would be to develop and run computations to determine most optimal conditions for maximizing production with this enzyme, as its properties have already been examined and reported.^26,49^

**Figure 9:**
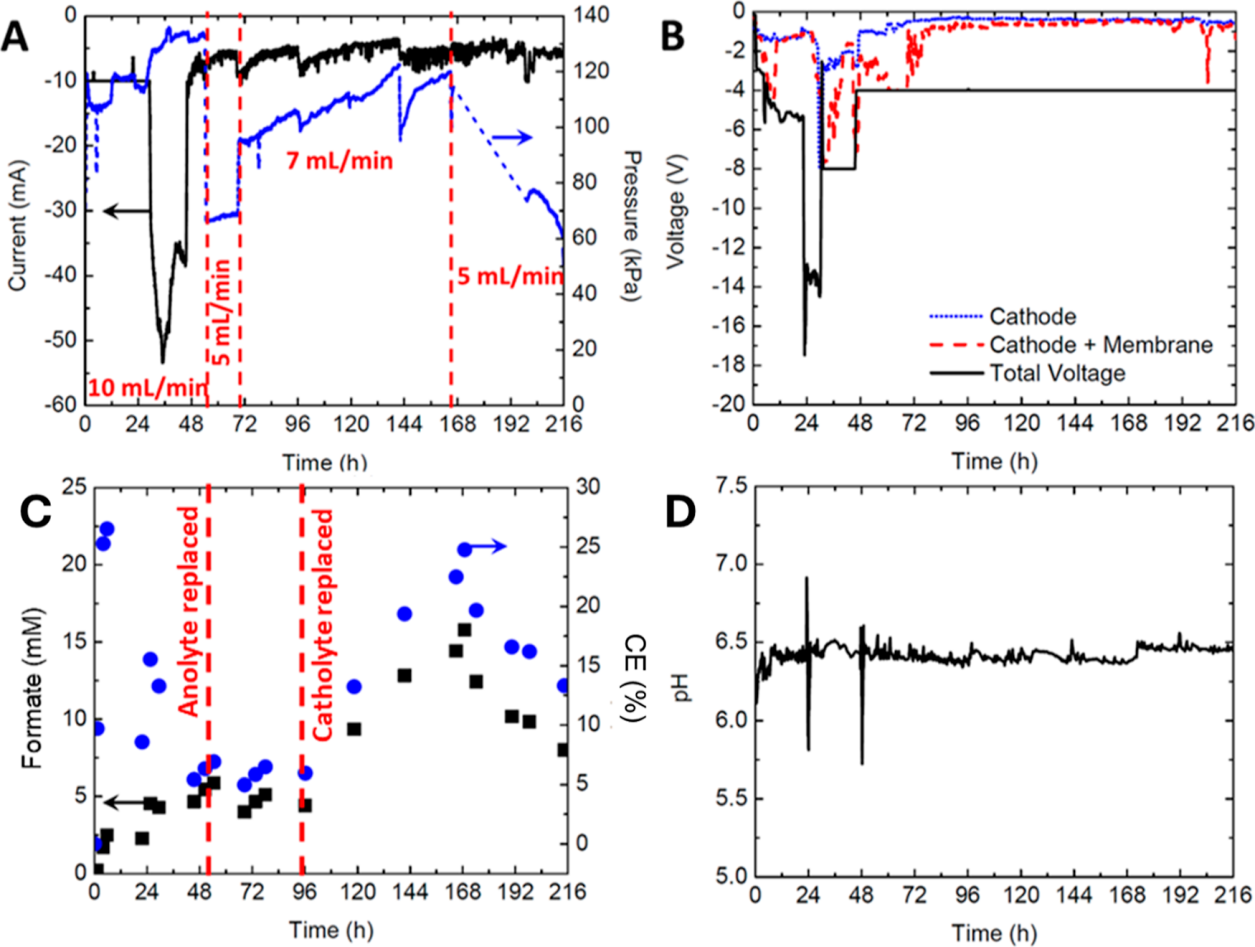
Packed bed reactor flow cell tests with pH control. (A) Current/pressure, showing varying flow rate, (B) voltage, (C) formate production/efficiency, and (D) pH. This experiment was run with variable applied voltages using 200 mM KHCO_3_ buffer in a cathode with 10 mM EV and 100 mM H_2_SO_4_ in the anode. The controls kept constant on the potentiostat during this experiment were −10 mA current for the first 30 h, −8 V total cell voltage for 30 to 48 h, and −4 V total cell voltage for the remainder of the experiment.

The packed bed reactor evaluated used an 11 cm column with 4 total enzyme samples (approximately 800 U total of activity) used in the system. To maintain a suitable pressure (<130 kPa gauge), the flow rate was adjusted during testing (Figure 9A). Reconfiguring the construction of the packed bed would be necessary to ensure that higher pressures can be accommodated. The continued pressure increase for a fixed flow rate suggests that there must be an accumulation of volume somewhere in the system and a steady-state pressure value has not yet been reached. The system should be analogous to an equivalent electrical circuit where the system flow rate remains constant.^50^ The pressure rise would have to be examined more rigorously by using increased system pressure capabilities to determine when the steady-state pressure is reached. A possibility is that gas evolution in the cathode is getting trapped in the production cell, which has been noted to increase pressure,^51−53^ particularly in battery systems.^54,55^ The gas evolution could also be verified using gas chromatography. Alternative system designs can also be considered to alter the pressure for this increase, and this has the added benefit of assisting in the CO_2_ conversion.^56^

Another change employed was setting a constant voltage on the entire cell instead of the cathode voltage only. Previous results have demonstrated challenges of keeping either the current or cathode voltage constant. If current is constant, required voltage in the production cell may not be high enough and can lead to decreases in performance. Constant cathode voltage is useful for formate production but may eventually destabilize since larger total cell voltages are used. Cell cutoff voltage was initially kept at −8 V to ensure that the cathode voltage would sufficiently reduce EV^2+^ but was lowered to −4 V as CO_2_ reduction cells generally recommend lower cell voltages to mitigate unwanted side reactions, electrode degradation, and excess energy consumption.^57−59^ At −4 V, the cathode voltage hovered around −0.6 to −0.7 V vs Ag/AgCl as measured, which still appeared sufficient to reduce EV^2+^ with the desired operating conditions in the new configuration (Figure 9B). Therefore, the new configuration aided in the production of formate while not needing to apply voltages as large. The ohmic drop across the membrane remained small over time (Figure 9B, red/blue lines). Therefore, nearly 75% of the total voltage in the cell was exclusively on the anode side, indicating that improvements could still be made on this side of the cell. Consideration must be taken that excess H_2_SO_4_ does not significantly decrease the pH of the cathode solution too quickly. Additionally, upon system scale-up, environmental factors regarding the effective use and disposal of H_2_SO_4_ must be factored in.^60^

The implemented changes improved stability initially, although the anode still contributed to most of the cell’s voltage (Figure 9B). As the voltage was lowered from −8 to −4 V in the early stages of the experiment, it was assumed that the excess voltage in the anode had already compromised the setup. Thankfully, the design of the dual-cell system enabled anode replacement without interrupting the cathode side or the packed bed. Solution replacement began when no EV^+^ was present, at approximately 48 h, indicated by the lack of blue coloring. During this time, the pump and potentiostat were turned off, the reduction cell was bypassed, and the cell was subsequently opened to replace the electrode on the anode side. Additionally, as a precaution, the system flow rate was temporarily decreased to 5 mL/min, before being restored to 7 mL/min for most of the remainder of the experiment (Figure 9A).

The anolyte replacement alone was insufficient to restore formate production, as samples taken in the next 48 h did not substantially increase. In the catholyte, overreduction to EV^0^ still occurred despite the lower applied voltage. This was evidenced by the appearance of a light-yellow coloration instead of the characteristic blue for EV^+^ (Figure S6), and its reversal is difficult. This EV^0^ formation could be due to the excessively high voltage in the system, or a result of other products forming within the catholyte, which may complicate the availability of the cathode to successfully reduce EV^2+^, or of the enzyme to reproduce it if formate production is stalled. pH is ruled out as a factor due to the buffer’s presence, although this value may not be consistent at all points within the system. The EV reduction becomes a problem as EV^0^ formation in solution is difficult to reverse and will hinder the enzyme’s ability to convert CO_2_ and H^+^ into HCOO^–^. Varying the total charge carrier in solution initially may serve as one way to mitigate this issue, along with alternative means through which excess charge can be dissipated when the cathode voltage may begin to produce EV^0^. Inevitably, maintaining the concentration needed to produce EV^+^ can become challenging, particularly when larger quantities of formate can begin to be produced. Mitigating this issue along with maintaining the system voltage and pH can enable formate production to remain at the levels seen in Figure 3.

With the voltage increase at nearly 24 h factored in, this led to a sharp increase in input energy (Figure S7), leading to lower energy efficiencies than those seen in previous studies. The peak EE in the early stages of the experiment, however, still approached 40%, rivaling the studies for the original system configuration seen in Figure S5.

The cathode solution was also replaced to eliminate excess EV^0^ and reset the catholyte with new EV^2+^ in the solution. This replacement was carried out later, at 96 h, only with the anode side of the cell kept intact and the cathode side circulated to empty the existing solution and enable new solution in the bulk volume. After the second replacement at 96 h, the enzyme was able to continue producing formate (Figure 9C). However, at around 168 h, the catholyte began to demonstrate excess EV^0^ formation again, suggesting the continuous need for replacement within the system.

Ultimately, both solutions would need to continuously be replenished due to the presence of unwanted side reactions and eventual deterioration of both electrodes; here, only one replacement of both solutions was shown. It should be noted that this issue would only arise in an experiment where a finite bulk volume is constantly being recycled (likely once every 48–72 h indicated by the production trends in Figure 9C). Thus, the long-term use of the flow system must carefully regulate the cell voltage such that EV^0^ production is kept minimal. The recovery of EV is important to consider during the future scale-up of this type of system, and the formation of EV^0^ would need to be carefully quantified. Additional experimentation on the system would be needed to pinpoint this issue, presumably first in the absence of an enzyme in the flow system before putting one in and then observing the subsequent effect on the voltage. In future studies, it must be a key focal point to ensure that the system can operate continuously and reliably produce formate at reasonably high efficiencies. System redesigns and possible catholyte additives should be examined as needed to either reduce or eliminate unwanted reactions, such as this EV^0^ formation or the dimerization of the charge carrier.

With pH control implemented, the desired pH range (6.3–6.8) could be maintained (Figure 9D). It should be noted that as the buffer diluted the cell volume, this diluted the concentration of the charge carrier and formate produced. However, the increased volume was factored into the total number of moles produced for efficiency. The formate and efficiency results (Figure 9C) depict both benefits and the drawbacks of using the system with the packed bed reactor. In the early stages of the experiment (<40 h), where cell current/voltage is maximized, more formate is produced and efficiency exceeds 25%. While lower than previous results from Figure 3, the configuration introduced new challenges, most notably the gradual increase in pressure even with a constant flow rate. Even when the total cell voltage was decreased, formate production stagnated, leading to diminished efficiency values (Figure 9C, 24–48 h). Furthermore, the inability to increase formate production is likely a result of the reduction cell overcharging and conversion of EV^+^ to EV^0^ (a characteristic yellowish tint was seen in the solution at this point in the experiment, Figure S6) and anode instability, since the Pt–Ir electrode on carbon was shown to destabilize over time when exposed to a sufficiently high charging voltage (Figure S8).

With both applied voltage and flow rate decreased and the solutions replaced, formate production retained nearly the same initial efficiency and it increased production to over 15 mM, a significant amount after considering the larger bulk volume in the flow system. Due to the implementation of the pH buffer and increased volume, the number of moles is increased with the peak formate production and cell volume increased to a 100 mL maximum. The number of moles produced increased to 3.6 mmol, the highest level obtained from this work.

When the flow rate in the system decreased, EV^2+^/EV^+^ conversion was hindered, resulting in an insufficient charge mediator for the enzyme. Nevertheless, the enzyme remained stable as production around the second peak (∼168 h) was comparable to the value in which the first peak (∼10 h) had occurred. Therefore, it can be concluded that management of both cathode solution and flow rate/pressure is crucial to maintaining formate production in a recirculating flow system. For further improvement, the volume of the cathode vessel can be increased to delay the excess reduction of EV^+^ to EV^0^, with a higher flow rate to decrease the residence time. Operating near the standard potential could also be made more feasible, to decrease charge input and then raise efficiency. This would require a larger mass of enzyme to maintain concentration, which would again suggest redesigning the packed bed reactor. If higher pressure could be accommodated, it could increase formate production due to the higher solubility of CO_2_.

In summary, we report on the unique design and construction of a dual-cell flow system that electrochemically converts CO_2_ to formate first using an electrochemical reduction of a charge carrier, then having said reduced charge carrier reoxidize with an enzymatic catalyst to produce HCOO^–^. The two processes are carried out in separate cells within the reactor. Different design considerations are discussed, including the effect of sampling location for measuring formate, the effect of applied cathode voltage, and the implementation of a pH control system to sustain pH and enable greater contact with the enzyme to continue producing formate long-term. While various challenges during each study were encountered, this is nonetheless a first of its kind configuration that demonstrates successful conversion to formate and can be implemented into a range of different systems.

## Conclusions

A dual-cell flow reactor has been developed to electrochemically reduce a viologen charge mediator and then use the said mediator to activate an enzyme to selectively convert CO_2_ to FA in the form of formate. This configuration is unique and design alternatives to sustain and improve the system’s performance are presented here. The experimentally designed system produced a maximum of 25 mM and reached efficiencies of over 50%. It was shown that if immediately extracted from the bulk solution, the increased formate concentration increases by 20% on average, due to dilution within the bulk solution. The transit time in between the reduction and production cells indicates that higher voltages to the system can be applied, greater than over −1 V vs Ag/AgCl. To boost the system’s long-term performance, two design changes are implemented: Introduction of the pH buffer to ensure that the enzyme would remain active, and replacement of the production cell with a packed bed reactor to further improve contact during the formate production process. The combined effects increased long-term formate production and efficiency, although adjustments to the flow rate and bulk solutions on each side of the system were needed to maintain this. This would allow the system to sustain larger production rates and efficiency.

## Supporting Information Available

Comparison between copper and carbon felt electrodes for the cathode, sizes for different membrane pores and catalyst components, illustration of the catalyst’s activity to convert CO_2_ and protons into formate, schematic of the pH control pump setup, EE for all experimental runs, picture of the in-lab setup of the bulk solution containing catholyte, plots of energy accumulation and EE for the dual-cell reactor, and comparison of different anode materials (PDF)
