# Supplementary material for: Decoupling Charge Carrier Electroreduction and Enzymatic CO2 Conversion to Formate Using a Dual-Cell Flow Reactor System
Source: ACS Omega. 2024 Sep 9;9(38):39353–64. doi: 10.1021/acsomega.4c02134 (PMC11425623; doi:10.1021/acsomega.4c02134)
Supplement: Supplementary file 1 — ao4c02134_si_001.pdf [file ao4c02134_si_001.pdf]

Decoupling Charge Carrier Electroreduction and Enzymatic CO_2 Conversion to Formate Using a Dual-Cell Flow Reactor System

Daniel Moreno [1], Ayokunle Omosebi [2], Byoung Wook Jeon [3], Keemia Abad [2,4], Yong Hwan Kim [3], Jesse Thompson [2,4], Kunlei Liu [5]

1. Missouri State University, Springfield, MO, USA
2. Institute for Decarbonization and Energy Advancement, University of Kentucky, Lexington, KY, USA
3. Ulsan National Institute of Science and Technology, Eonyang-eup, Ulju-gun, Ulsan, South Korea
4. Department of Chemistry, University of Kentucky, Lexington, KY, USA
5. Department of Mechanical and Aerospace Engineering, University of Kentucky, Lexington, KY, USA

* **Corresponding Author:** Dr. Daniel A. Moreno, Assistant Professor, Mechanical Engineering, Cooperative Engineering Program at Missouri State University, PCTR 2021G · 405 N. Jefferson Ave. · Springfield, MO 65897, 417-837-2329 · danielmoreno@missouristate.edu

Supporting Information:

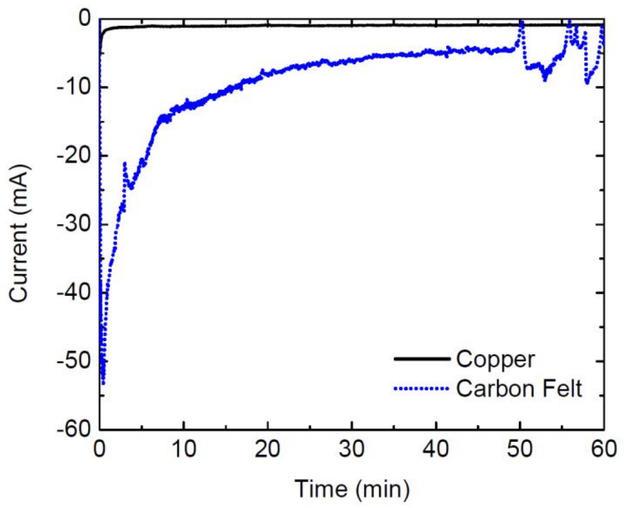

Figure S1. Current data for a flow system tested using only the reduction cell, to determine the influence of the cathode material. Cathode consisted of 10 mM methyl viologen in 200 mM potassium phosphate buffer solution (50/50 monobasic/dibasic), anode consisted of a platinum mesh electrode immersed in 1 mM H_2SO_4. Applied reduction cell voltage was -0.75 V vs. Ag/AgCl, with both anode/cathode solution volumes at 40 mL circulated at 10 mL/min. Electrode size was 1.5 cm^2 for both electrodes, indicating the advantages of the porous carbon felt.

Table S1. Approximate sizes for different membrane pores and catalyst components.

Catalyst (estimated)	>75 kDa
Tri-Sep UF 5	5 kDa
Tri-Sep UF 10	10 kDa
Synder MQ	50 kDa
Snyder PZ	30 kDa
Agarose Bead + Catalyst	>150 μm
Mesh Membrane	100 μm

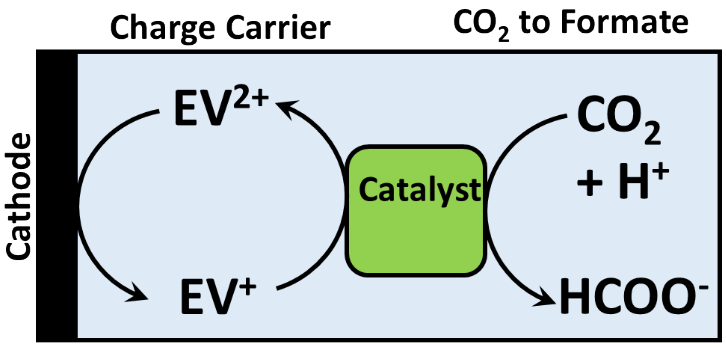

Figure S2. Illustration of the catalyst's activity to convert CO_2 and protons into formate.

The pH controller and pump (Hanna Instruments, BL7916) was used to maintain the pH in the cathode compartment of the flow cell by gradual dosing of potassium hydroxide (KOH) To decrease the required titrant volume in the cell, the reservoir (Figure S3) was set to pH = 12. The feedback loop implemented in the controller would slow down pumping as pH approaches the set point, to minimize the dosing of excess pH and causing problems with overflow and an imbalance of pH throughout the entire reactor. By using an appropriate resistor configuration in the pump's control panel, including a potentiometer to adjust as needed, flow control could be tuned to slow down flow if pH is as much as 2 units below the set point, which was important to consider due to small system volume relative to the pump. Here, the set point selected was 6.5, to ensure that pumping would not stop close to the set point. A recirculation branch is also used to minimize the KOH that is pumped into solution to prevent overflow and overconcentration, as well as a pressure accumulation. A pressure gage ensures that pumping pressure does not exceed the tubing limit of 50 psi (gage). In normal batch cell operation, the pressure did not go beyond 5 psi. The procedure for operating the pump unit as follows:

1. Set up reactor cell as desired.
2. Set up pH in reservoir.
3. Fully close valve leading into reactor.
4. Select desired pH set point.
5. Turn on pump to ensure proper operation.
6. Start experiment.
7. Open reactor valve gradually to ensure small base volume pumped into solution as needed.

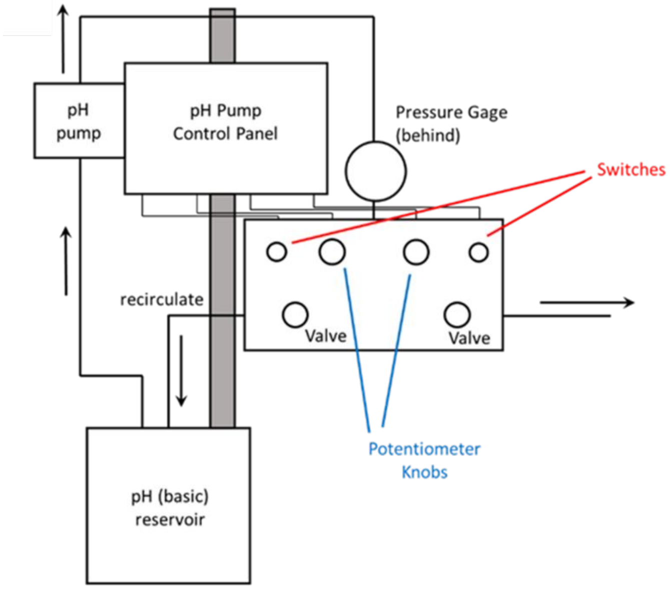

Figure S3. Schematic of pH pump control setup.

Energy Efficiency:

Energy Efficiency (EE) was calculated using the following equation (1):

$$EE = \frac{|n_{HCOO^-} \Delta G_{f,HCOO}|}{\int VI\, dt}$$

The denominator essentially represents the total electrical energy input during the experimental running time. In the numerator, n_{HCOO^-} represents the total number of moles for formate produced (molar concentration x solution volume), and $\Delta G_{f,HCOO}$ represents the Gibbs free energy of formate, here estimated at -160 kJ/mol. This assumed the cell required a potential of -0.83 V vs. Ag/AgCl to react, with 2 electrons required per interaction to produce formate.

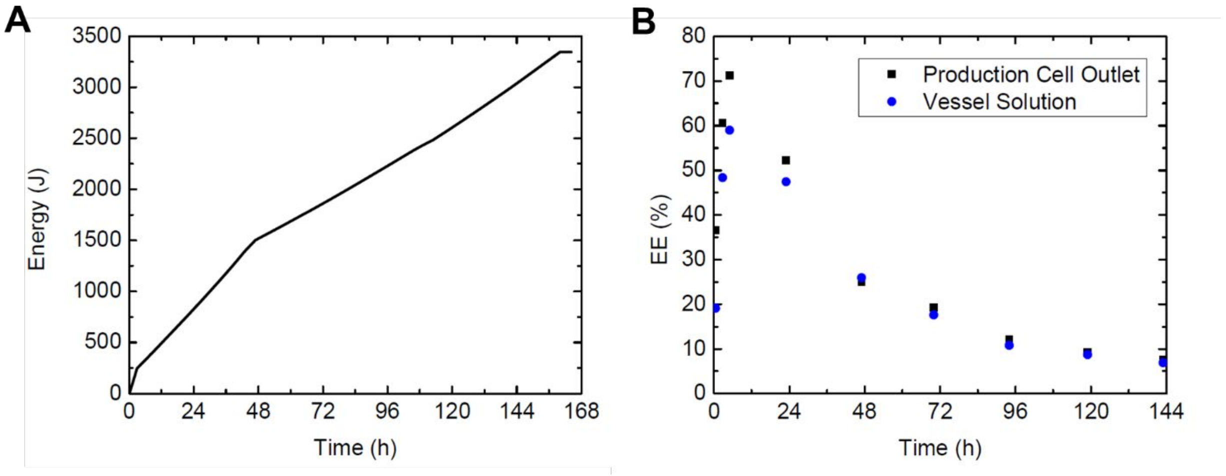

Figure S4. (A) Plot of energy accumulation for original dual-cell reactor configuration, (B) plot of energy efficiency based on formate values obtained at different locations.

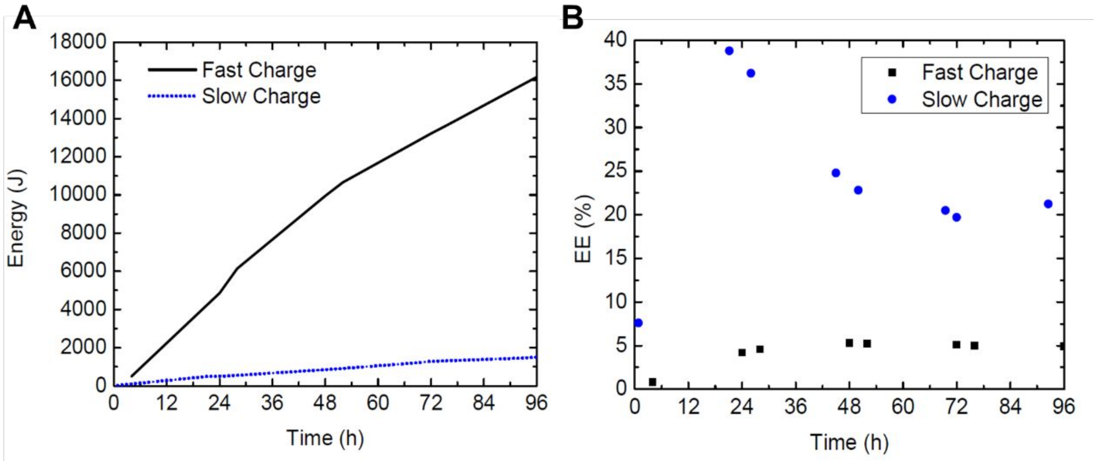

Figure S5. Plots of (A) energy accumulation and (B) energy efficiency at different reactor charging rates.

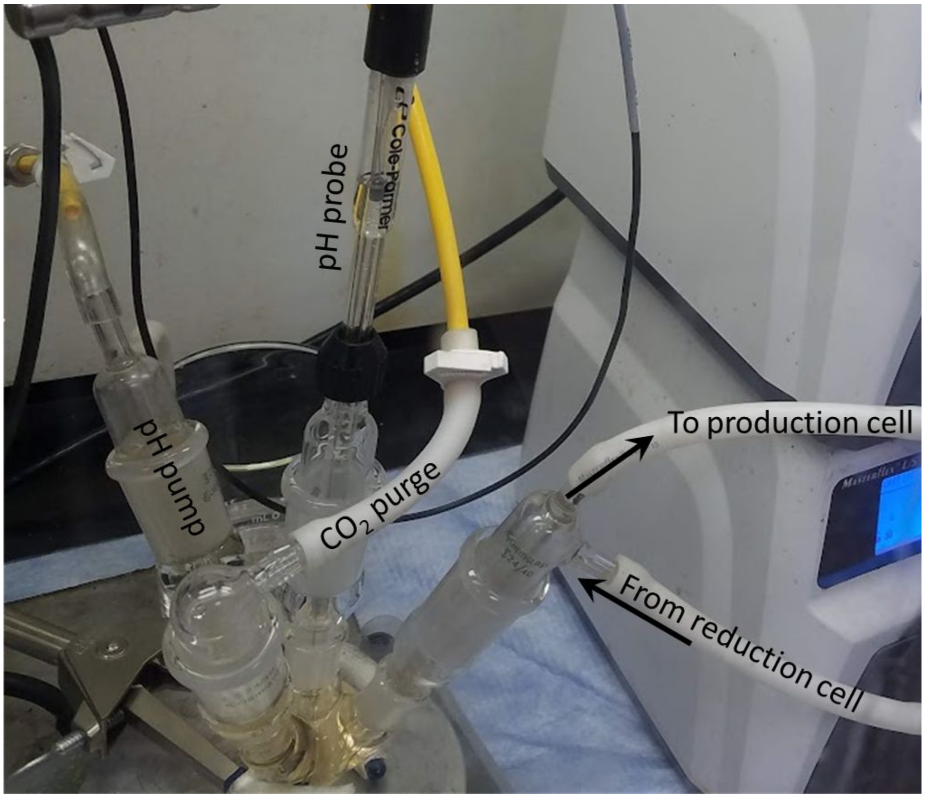

Figure S6. Setup of bulk solution containing catholyte in lab, indicating flow between cells, with pumps shown at the right, and yellow liquid coloration indicating overreduction.

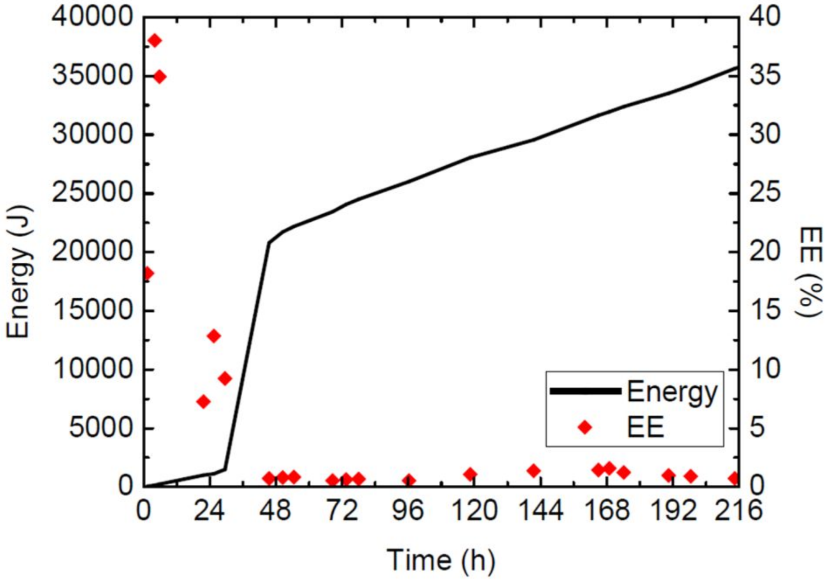

Figure S7. Plots of energy accumulation and energy efficiency for the dual-cell reactor with the packed bed configuration.

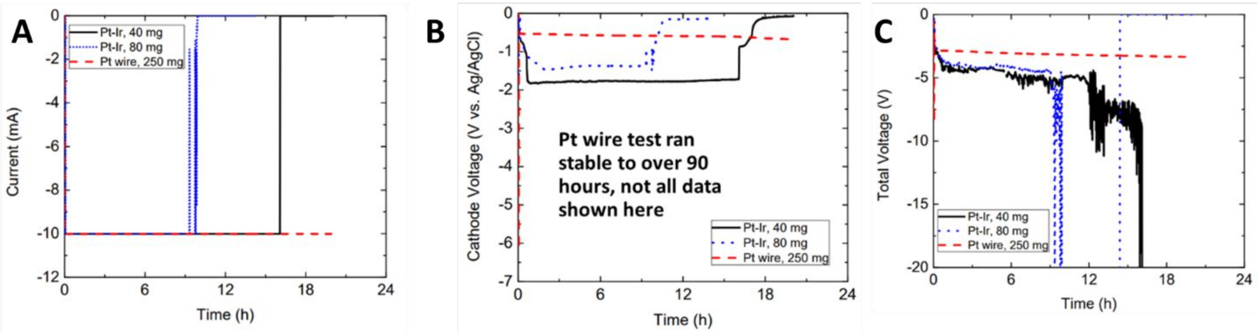

Figure S8. Comparison of different anode materials – wire and cloth with different coated masses on carbon. (A) Current, (B) Cathode voltage, (C) Total voltage. Tests were run in a 10 mL batch cell, with 100 mM H_2SO_4 in the anode and 200 mM $KHCO_3$ in the cathode. Cathode used a 1.5 cm^2 carbon felt electrode of 180 mg.

References:

1. Morrison AR, van Beusekom V, Ramdin M, van den Broeke LJ, Vlugt TJ, de Jong W. Modeling the electrochemical conversion of carbon dioxide to formic acid or formate at elevated pressures. Journal of The Electrochemical Society. 2019;166(4):E77.
